# Long-term incidence and progression of vision-threatening diabetic retinopathy in Asian populations

**DOI:** 10.7189/jogh.16.04174

**Published:** 2026-06-19

**Authors:** Barry Moses Quan Ren Koh, Crystal Chong, Gavin Tan, Ching-Yu Cheng, Charumathi Sabanayagam

**Affiliations:** 1 Singapore Eye Research Institute, Singapore National Eye Centre, Singapore; 2 Ophthalmology and Visual Sciences Academic Clinical Programme, Duke-NUS Medical School, Singapore

## Abstract

**Background:**

To evaluate incidence, progression and risk factors of vision-threatening diabetic retinopathy (VTDR) in two Asian populations with diabetes.

**Methods:**

We included data from 1177 Malay and Indian adults aged 40–80 years with diabetes, who participated in baseline (2004–2009) and 12-year follow-up (2017–2023) of the Singapore Epidemiology of Eye Diseases study (SEED). Incident VTDR was defined among participants free of any DR at baseline as the development of severe non-proliferative DR, proliferative DR, or clinically significant macular oedema at follow-up, assessed from two-field fundus photography using ETDRS severity scale without optical coherence tomography (OCT). Progression was assessed in those with mild or moderate DR at baseline (n = 211). Risk factors included age, sex, ethnicity, systolic blood pressure (BP), duration of diabetes, and glycated haemoglobin (HbA1c). Associations between risk factors and outcomes were evaluated using Poisson regression models, and results presented as relative risks (RR) with 95% confidence intervals (CI).

**Results:**

The 12-year cumulative incidence of VTDR was 3.8% (Indians = 4.4%; Malays = 3.0%) while progression to VTDR was 17.1% (Indians = 18.4%; Malays = 14%). In multivariable models, Indian ethnicity (RR = 2.23; 95% CI = 1.17–4.27), higher systolic BP (RR = 1.02; CI = 1.00–1.03), longer duration of diabetes (RR = 1.05; CI = 1.00–1.09), and higher HbA1c (RR = 1.52; CI = 1.35–1.70) were positively associated whereas older age (RR = 0.95; CI = 0.91–0.99) was negatively associated with incident VTDR. Higher HbA1c (RR = 1.29; CI = 1.11–1.49) was associated with VTDR progression.

**Conclusions:**

In this 12-year population-based study, we found a low cumulative incidence of VTDR (4%) but a relatively high progression among those with baseline DR (17%). These findings support the potential value of risk-stratified screening approaches. Individuals without DR at baseline may be considered for less frequent screening, whereas those with mild DR and/or poor glycaemic control may benefit from closer monitoring due to their higher risk of progression.

Diabetes currently affects approximately 460 million individuals globally and this is expected to rise to 578 million by 2030 [1]. Progression of diabetic retinopathy (DR) can result in vision threatening diabetic retinopathy (VTDR) which comprises severe non-proliferative DR, proliferative DR, and diabetic macular oedema. In 2020, there were 29 million individuals with VTDR, and this is projected to increase to 45 million by 2045 [2]. With progression to VTDR, studies have shown that not only does it result in vision loss but also in increase in societal burdens, decrease quality of life [3], increased risk of cardiovascular diseases and mortality [4].

To date, most epidemiological research on DR have focused on its prevalence with limited data on the incidence and progression of DR. Asians have been identified as high-risk group for diabetes. In Singapore, Malays and Indians are particularly at high risk, with a diabetes prevalence exceeding 14% in the 2020 National Population Health Survey. Cross-sectional data from our Singapore Epidemiology of Eye Diseases (SEED) study showed that the prevalence of DR was higher in Indians compared to Malays and Chinese despite similar risk factor profiles across these populations [5]. In a separate longitudinal study from our group, the 6-year incidence of DR was comparable between Malays and Indians, with rates exceeding 18% in both populations. However, the 6-year incidence of proliferative DR was lower in Indians compared to Malays (0.2% *vs*. 1%). Similarly, progression to proliferative DR was also lower in Indians (1%) compared to Malays (2%). These findings highlight significant ethnic disparities in the development, and progression of advanced stages of DR, such as proliferative DR, highlighting the need for further investigation to understand these differences and inform targeted interventions. With the availability of 12-year follow-up data, our study offers a rare opportunity to examine the long-term incidence and progression of VTDR, as well as to explore ethnic differences over an extended period. This substantially advances prior work, which has largely been cross-sectional or based on shorter follow-up durations.

In this context, we aimed to evaluated the 12-year incidence of DR and VTDR and progression to VTDR among Malay and Indian participants who completed the 12-year follow-up study. By leveraging a population-based, multi-ethnic cohort with standardised retinal grading, our study provides robust long-term evidence to better characterise ethnic disparities and risk profiles for VTDR.

## METHODS

### Study population

Data for this study was derived from two prospective cohort studies: the Singapore Malay Eye Study (SiMES) and the Singapore Indian Eye Study (SINDI). Data for this study were derived from two prospective cohort studies of Malay and Indian adults: the Singapore Malay Eye Study (SiMES) and the Singapore Indian Eye Study (SINDI). Participants aged 40–80 years at baseline who attended both the baseline (2004–2009) and follow-up (2011–2015) examinations were included (n = 6680).

Of these, individuals without diabetes (n = 3968), those with type 1 diabetes (defined as diabetes onset before 30 years of age; n = 17), and those lost to follow-up or with missing DR grading (n = 1244) were excluded, resulting in a final sample of 1451 participants with presumed type 2 diabetes. From this group, participants with prevalent DR at baseline (n = 274) were further excluded, leaving 1177 individuals (499 Malay, 678 Indians) for the incident DR analysis. Progression was evaluated in those with mild or moderate DR at baseline (n = 211). The study methodology and details have been previously reported [6]. Informed written consent was obtained from all participants, and ethical approval was obtained from the Institutional Review Board of the Singapore Eye Research Institute. The Singapore Epidemiology of Eye Diseases study was conducted in accordance with the tenets of the Declaration of Helsinki and ethics.

### Retinal imaging

Two-fields retinal photographs (macula-centred and optic disc-centred) were obtained from both eyes of each participant using a 45° non-mydriatic digital retinal camera (Canon CR-DGi with a 10D/20D SLR backing, Canon, Japan in SEED) and graded for DR by trained professional graders. Retinopathy was considered to be present if any characteristic lesion as defined by the Early Treatment Diabetic Retinopathy Study (ETDRS) severity scale was present, and retinopathy severity was assigned according to the modified Airlie House Classification system [7]. Diabetic retinopathy grading was based solely on two-field fundus photography, without optical coherence tomography (OCT). This approach may underestimate peripheral lesions and macular oedema, potentially leading to under-ascertainment of VTDR.

Based on the severity score of the worse eye, incident DR was defined as development of any new DR (defined as score level 20 and above in at least one eye) at the follow-up among those who had no retinopathy at baseline (ETDRS level 10/10 in both eyes). Our primary outcome, incident VTDR was defined by the presence of severe non-proliferative DR, proliferative DR or clinically significant macular oedema (defined as score levels 53 and above) [5] at the follow-up. These conditions represent the advanced stages of DR that pose the highest risk of irreversible vision loss and typically warrant similar levels of clinical attention and intervention. Progression to VTDR was defined as an increase in disease severity from mild or moderate DR at baseline to VTDR at follow-up. Although our primary focus was on the incidence and progression of VTDR, we also reported on any DR for completeness.

### Risk factor assessment

Risk factors considered included age, sex, ethnicity, education, smoking, body mass index (BMI), blood pressure (BP), estimated glomerular filtration rate (eGFR), total and HDL Cholesterol, duration of diabetes, and glycated haemoglobin (HbA1c). Presence of diabetes was defined as a random plasma glucose ≥11.1 millimoles per litre (mmoL)/L, HbA1c ≥6.5%, or participants self-reported use of anti-diabetic medication or had physician-diagnosed diabetes. Body mass index was calculated from measured height and weight (kg/m^2^) and BP measurements were taken during the clinical examination. Hypertension was defined as systolic BP>140 mm of mercury (mmHg), diastolic BP>90 mm Hg, or use of antihypertensive medication. All examinations followed standardised protocols. Non-fasting venous blood samples were taken and sent for analysis of serum lipid levels (total cholesterol, high-density lipoprotein (HDL) cholesterol, and low-density lipoprotein (LDL) cholesterol), HbA1c on the same day. Participants’ age, sex, ethnicity, education, smoking status, duration of diabetes, and sex were self-reported.

### Statistical analysis

Characteristics of the study population were evaluated stratified by incident VTDR and summarised as mean (standard deviation) for continuous variables or number (percent %) for categorical variables, with *P* values calculated using Student’s *t* test or χ^2^ test as appropriate for the variable.

**Figure 1:**
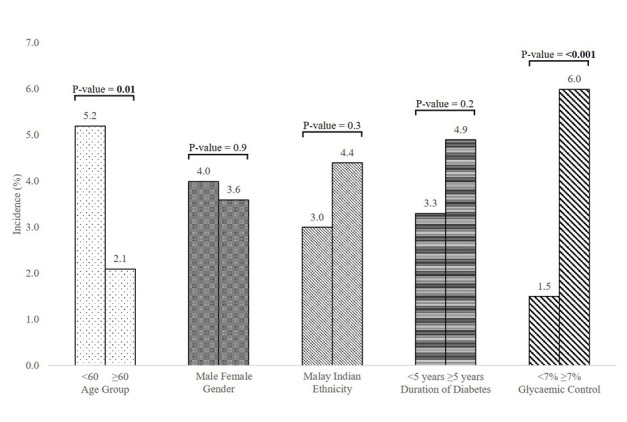
Incidence of VTDR by subgroups. VTDR – Vision-threatening diabetic retinopathy.

For the VTDR analyses, participants who did not develop any DR or developed only mild or moderate DR during follow-up were classified as controls (no VTDR). Risk factors were examined by comparing individuals with and without incident VTDR. As the primary objective was to examine the incidence and progression of VTDR, we reported overall incident DR to provide context but focused mainly on VTDR-related analyses. Although we had information on many potential risk factors such as education, smoking, BMI, and cholesterol profile, as the number of VTDR events was limited, we included only established risk factors for DR such as age, sex, ethnicity, duration of diabetes, systolic BP, and HbA1c in the multivariable models for both incident and progressive VTDR. Incidence was calculated as cumulative incidence, ie, incidence proportion from baseline to 12-year. In subgroup analysis, we stratified incident VTDR by age (<60 *vs*. ≥60 years), gender (male *vs*. female), ethnicity (Malay *vs*. Indians), duration of diabetes (<5 *vs*. ≥5 years) and glycaemic control (<7% *vs*. ≥7%). These differences were illustrated using bar charts (**Figure 1**).

Associations between risk factors and outcomes were evaluated using Poisson regression models, with results presented as relative risks (RR) with 95% confidence intervals (CI). Poisson regression was used to estimate RRs for cumulative incidence and progression of VTDR because outcomes were assessed only at discrete follow-up visits. A time-to-event approach (Cox proportional hazards modelling) was not feasible, as exact dates of VTDR onset and interval-censoring information were unavailable.

Model assumptions were examined to ensure appropriate model fit. Equidispersion was assessed using the ratio of residual deviance to degrees of freedom from an equivalent Poisson model (deviance/df = 0.21), indicating no evidence of overdispersion. Multicollinearity was evaluated using variance inflation factors from an equivalent generalised linear model, with all VIF values below 1.3, suggesting no problematic collinearity among predictors. In the current analysis, we excluded 1244 diabetic participants due to loss to follow-up or missing variables, which may have introduced selection bias. Therefore, in a separate analysis, we compared the baseline characteristics of excluded participants with those included in the analysis to assess the potential for selection bias.

Progression to VTDR was calculated as the cumulative progression over the entire follow up period. As the number of progression events was low (n = 25), we reported overall progression and associated risk factors without stratifying analyses by subgroups.

## RESULTS

**Table 1** summarises the baseline characteristics of the 1177 participants with diabetes stratified by incident VTDR status. Of these participants, 50.8% were males, 42.8% were Malays, while 57.2% were Indians. Mean age of participants without incident VTDR was 59.1 years and of those with incident VTDR was 55.3 years. Compared to participants without incident VTDR, those with incident VTDR were younger, had higher HbA1c, total cholesterol, eGFR, and lower BMI. Compared to participants without progression to VTDR, those with progression to VTDR had higher HbA1c.

**Table 1:** Characteristics of participants by incident VTDR status

	No incident VTDR (n = 1132)	Incident VTDR (n = 45)	*P*-value*
Age, years	59.1 (9.0)	55.3 (8.6)	0.005
Gender, %			
*Female,*	557 (49.2)	21 (46.7)	0.9
*Male*	575 (50.8)	24 (53.3)	
Ethnicity, %			
*Malay*	484 (42.8)	15 (33.3)	0.3
*Indian*	648 (57.2)	30 (66.7)	
Education level, %			
*Below primary education*	229 (20.2)	5 (11.1)	0.3
*Primary education*	476 (42.0)	19 (42.2)	
*Secondary/above education*	427 (37.7)	21 (46.7)	
Current smoking, yes, %	153 (13.5)	4 (8.9)	0.5
BMI, kg/m^2^	28.2 (4.9)	27.0 (4.5)	<0.001
Systolic BP, mmHg	141.3 (19.6)	145.0 (18.9)	0.2
Total cholesterol, mmol/L	5.1 (1.1)	5.7 (1.2)	0.002
HDL cholesterol, mmol/L	1.1 (0.3)	1.1 (0.3)	0.5
Duration of diabetes, years	4.4 (6.0)	5.0 (7.1)	0.5
HbA1c, %	7.5 (1.5)	9.2 (2.3)	<0.001
HbA1c categories			
*<7%*	540 (48.7)	8 (18.2)	<0.001
*7–8%*	292 (26.3)	8 (18.2)	
*≥8%*	277 (25.0)	28 (63.6)	

BP – blood pressure, BMI – body mass index, HbA1c – glycated haemoglobin, VTDR – vision threatening diabetic retinopathy

*P-values represent the difference in characteristics by incident VTDR status based on Student’s t-Test or χ2 test as appropriate for the variable.

### Incident VTDR and associated risk factors

The 12-year cumulative incidence of any DR was 22.3% (Indians = 21.8%; Malays = 23%, *P* = 0.7) and of VTDR was 3.8% (Indians = 4.4%; Malays = 3.0%). **Figure 1** shows the incidence of VTDR in subgroups. Incidence of VTDR was significantly higher in those aged <60 years compared to those ≥60 years (5.2% *vs*. 2.1%), and those with HbA1c >7% compared to ≤7% (6% *vs*. 1.5%). Although incidence was higher in males compared to females (4% *vs*. 3.6%), Indians compared to Malays (4.4% *vs*. 3%), and those with duration of diabetes <5 years compared to ≥5 years (3.3% *vs*. 4.9%), these differences were not significant (*P* > 0.1).

In multivariable models, Indian ethnicity (RR = 2.23; 95% CI = 1.17–4.27), higher systolic BP (RR = 1.02; 95% CI = 1.00–1.03), longer duration of diabetes (RR = 1.05; 95% CI = 1.00–1.09), and higher HbA1c (RR = 1.52; 95% CI = 1.35–1.70) were positively associated with incident VTDR. Older age (RR = 0.95; 95% CI = 0.91–0.99) was negatively associated with incident VTDR (**Table 2**). Education levels, smoking status, BMI, total and HDL cholesterol levels, eGFR, and use of anti-diabetic medication were not found to be significantly associated with incident VTDR (Table S1 in the **Online Supplementary Document**).

**Table 2:** Risk factors associated with Incident VTDR (No + mild + moderate DR *vs*. VTDR) in multivariable regression analysis

	Age, sex-adjusted RR (95% CI)*	*P*-value	Multivariable adjusted† RR (95% CI)*	*P*-value
Age, years	0.95 (0.92–0.99)	0.007	0.95 (0.91–0.99)	0.01
Gender				
*Male*	Reference		Reference	
*Female*	0.89 (0.50–1.57)	0.7	0.95 (0.54–1.67)	0.9
Ethnicity				
*Malay*	Reference		Reference	
*Indian*	1.45 (0.79–2.67)	0.2	2.23 (1.17–4.27)	0.02
Systolic BP, mmHg	1.01 (1.00–1.03)	0.02	1.02 (1.00–1.03)	0.02
Diabetic duration, years	1.04 (1.00–1.09)	0.1	1.05 (1.00–1.09)	0.05
HbA1c, %	1.46 (1.32–1.62)	<0.001	1.52 (1.35–1.70)	<0.001

BP – blood pressure, CI – confidence interval, HbA1c – glycated haemoglobin, RR – relative risk, VTDR – vision threatening diabetic retinopathy

*RRs were calculated using modified Poisson regression, with a robust error variance.

†Adjusted for age, sex, ethnicity, duration of diabetes, systolic BP and HbA1c.

In sex-specific analyses (Table S2 in the **Online Supplementary Document**), higher systolic BP and HbA1c were positively associated with incident VTDR in females, while older age and use of anti-diabetic medication were negatively associated. In males, only longer duration of diabetes and higher HbA1c were significantly associated with incident VTDR. In ethnicity-specific analyses (Table S3 in the **Online Supplementary Document**), higher systolic BP was positively associated with incident VTDR among Malays, whereas higher total cholesterol was associated with increased risk in Indians. Both elevated HbA1c and longer diabetes duration were positively associated with incident VTDR in both ethnicities. In analysis comparing the characteristics of excluded vs included participants (Table S4 in the **Online Supplementary Document**), we found that those who were excluded were older, had higher systolic BP, higher HbA1c, and longer duration of diabetes.

### Progression to VTDR and associated risk factors

At 12-year, 36 of the 211 participants with mild/moderate DR at baseline progressed to VTDR with a cumulative progression of 17.1% overall (Indians = 18.4%; Malays = 14%, *P* = 0.6). Progression was nearly four times higher from moderate DR compared to mild DR at baseline (39.2% *vs*. 10.9%). In multivariable model analysis, only higher HbA1c (RR = 1.29; 95% CI = 1.11–1.49) was associated with progression (**Table 3**). Other risk factors such as ethnicity, higher systolic BP, and longer duration of diabetes were not associated with progression to VTDR

**Table 3:** Risk factors associated with progression to VTDR in multivariable regression analysis

	Age, sex-adjusted RR (95% CI)*	*P*-value	Multivariable adjusted† RR (95% CI)*	*P*-value
Age, years	1.01 (0.97–1.04)	0.7	1.02 (0.97–1.07)	0.4
Gender				
*Male*	Reference		Reference	
*Female*	1.14 (0.63–2.09)	0.7	0.94 (0.50–1.75)	0.8
Ethnicity				
*Malay*	Reference		Reference	
*Indian*	1.34 (0.66–2.71)	0.4	1.77 (0.83–3.79)	0.1
Systolic BP, mmHg	1.00 (0.99–1.02)	0.9	1.00 (0.99–1.02)	0.6
Diabetic duration, years	1.01 (0.97–1.05)	0.5	0.99 (0.95–1.04)	0.8
HbA1c, %	1.25 (1.09–1.43)	0.002	1.29 (1.11–1.49)	<0.001

BP – blood pressure, CI – confidence interval, HbA1c – glycated haemoglobin, RR – relative risk, VTDR – vision threatening diabetic retinopathy

*RRs were calculated using modified Poisson regression, with a robust error variance.

†Adjusted for age, sex, ethnicity, duration of diabetes, systolic BP and HbA1c

## DISCUSSION

In this population study of a multi-ethnic Asian cohort aged 40 to 80 years followed over 12 years, the cumulative incidence of VTDR was 3.8% (Indians = 4.4%; Malays = 3.0%). Progression to VTDR among those with baseline DR was 17.1% (Indians = 18.4%; Malays = 14%). Indian ethnicity, longer duration of diabetes, higher systolic BP, and higher HbA1c levels were associated with incident VTDR. Higher HbA1c was associated with VTDR progression, while the use of anti-diabetic medication was associated with a lower risk of progression.

In this study, the 12-year cumulative incidence of VTDR was 3.8%. This is comparable to the 4.5% reported in the Multi-Ethnic Study of Atherosclerosis (MESA) in the USA, which included 2.75% for incident VTDR (including NPDR and clinically significant macular oedema (CSME)) and 1.8% for PDR) [8]. Of note, MESA separated PDR from VTDR, while our study combined the 2 groups. Our study’s finding is lower than the 8.3% reported over nine years in Barbado’s Incidence Study of Eye Diseases (BISED) II cohort study of 436 participants. Compared to regional Asian studies, the incidence of VTDR in our study was higher. For example, in India’s Sankara Nethralaya-Diabetic Retinopathy Epidemiology and Molecular Genetic Study (SN-DREAMS) 15-year follow-up study, the reported cumulative incidence of VTDR was 1.1% [9–12]. The higher incidence observed in our study compared to the Indian SN-DREAMS study may be attributed to several factors. First, the use of systematic screening efforts in our cohort could have led to increased detection of VTDR cases. Additionally, lifestyle factors associated with a high-income, urbanised setting such as sedentary behaviour and dietary changes may contribute to a higher risk of VTDR. Methodological differences may also have played a role; for example, SN-DREAMS employed a simpler DR grading (International Clinical Diabetic Retinopathy Severity Scale), whereas our study used the more detailed ETDRS grading system, potentially leading to more accurate classification of disease severity.

There is little available data from Asia directly comparing the incidence of VTDR between ethnic groups. In multivariable analysis, Indian ethnicity was associated with VTDR with RR of 2.23 (95% CI = 1.17–4.27) compared to Malay ethnicity. Established risk factors namely higher BP, cholesterol, and HbA1c were associated with VTDR in both populations [5]. This is noteworthy, as in diabetic macular oedema (DME, a form of VTDR), lipid leakage from damaged retinal capillaries results in hard exudates and previous studies have shown a strong association between serum cholesterol levels and the severity of hard exudates and DME. Moreover, clinical trials such as FIELD [13] and ACCORD-Eye studies [14] have shown that fibrates, a lipid-lowering agent which reduce triglycerides, significantly reduce the progression of DR.

The progression to VTDR over 12 years was 17.1%. This progression is higher compared to developing countries such as India (1.75%%), Barbados (6.90%) [11], and Kenya (0%) [15], but aligns closer with findings with GDESP from the UK (13.4%) [16]. It has been speculated that increased acculturation to a westernised lifestyle associated with increased prevalence of obesity and diabetes has led to a higher progression of incident VTDR [17]. This may also explain the higher incidence and progression to VTDR seen in Indians in Singapore compared to ethnic Indians in the SN-DREAMS cohort.

Another possible explanation could be that studies with a longer duration of follow up (SN-DREAMS 15 years follow-up; BISED II 9 years follow-up) might have higher mortality. For example, in Kenya’s Nakuru cohort study [15], the progression is reported as 0%, which may reflect limited follow-up or high mortality. Owing to significant advancements in diagnosis technology and risk factor management for DR in the past 20 years, progression rates reported previously may not reflect current rates. This may also explain the lower incidence reported in these countries as well.

### Factor associated with VTDR incidence and progression

In multivariable model, Indian ethnicity and longer duration of diabetes (non-modifiable risk factors) as well as higher HbA1c and systolic BP (modifiable risk factors), were found to be positively associated with incidence of VTDR. This finding aligns with the majority of studies showing that longer duration of diabetes is a key risk factor for incident DR [14,18], and that higher HbA1c levels and elevated systolic BP are positively associated with the development of VTDR [16,19,20]. Higher HbA1c indicating poor glycaemic control is an established risk factor for DR [21]. Duration of diabetes is a known risk factor for DR progression with long-term cohort studies with lengthy follow-up times finding that most patients with diabetes develop some degree of retinopathy given long enough exposure [22,23]. We found older age to be negatively associated with incident VTDR. This was likely due to the competing risk of mortality in patients with type 2 diabetes, who may have more age-related comorbidities [24].

Progression to VTDR was primarily associated with higher HbA1c levels. Other factors such as ethnicity, higher systolic BP, and longer duration of diabetes were not significantly associated with progression. This lack of association may be due to the relatively small sample size (n = 211 assessed for progression), which may have limited the statistical power to detect significant relationships. Both UK Prospective Diabetes Study (UKPDS) and the Diabetes Control and Complications Trial (DCCT) provided strong evidence that tight control of HbA1c <7% reduces the risk for progression of DR [20,25]. Progression of DR is heavily influenced by glycaemic variability and systemic metabolic control. Medications help stabilise these factors, slowing retinal damage [17]. The UKPDS and DCCT studies demonstrated that tight glycaemic control significantly associated with progression to VTDR, with mechanisms potentially linked to ‘metabolic memory [19,26].

Based on this cohort study, several strategies for VTDR management should be emphasised. First, to address the issue of the growing incidence of VTDR, additional studies representative of developed countries need to better characterise the incidence of VTDR. Second, innovative approaches to improve screening, such as AI screening tools and hand-help retinal cameras may enhance early detection. Third, of concern is that a large proportion of diagnosed DR is vision threatening. These observations imply that many cases have been detected late, when the disease has already progressed to a vision-threatening stage, or that these populations may be particularly susceptible to severe DR due to ethnic predisposition. Screening programmes could benefit from tailoring follow-up strategies based on distinct risk factor profiles for patients at risk of incident VTDR *vs*. those monitored for progression. Individuals at elevated risk for incident VTDR may require earlier initiation of screening, whereas those at higher risk of progression may need closer monitoring with shorter review intervals. Subgroups such as Indian participants, individuals with poor glycaemic control, and those with long-standing diabetes may particularly benefit from more frequent retinal examinations while those with stable metabolic control may be suitable for longer screening intervals. Incorporating systemic risk factors into screening algorithms could improve cost-effectiveness and optimise resource allocation. Fourth, future studies should adopt a standardised definition of VTDR in their methodology (*e.g*. some studies separate proliferative DR, diabetic macular oedema, and severe non-proliferative DR, which might dilute the estimates of incident VTDR).

This study has limitations. DR and DME were assessed using two-field fundus photography, which may have missed peripheral lesions. Additionally, optical coherence tomography (OCT) was not used to assess DME, which may have led to underestimation of DME and, consequently, VTDR incidence. Lost to follow-up from various causes such as death is particularly relevant in diabetic patients who are at higher risk of death from comorbidities, potentially causing selection bias from underestimation of the true estimates of incidence and progression. In our study, excluded participants were older at baseline, suggesting the possibility of competing mortality. They also had higher systolic BP, higher HbA1c, and longer diabetes duration, indicating that the analytic cohort may represent a healthier subset of the original population. Consequently, absolute incidence and progression rates may be underestimated, and some risk associations may be attenuated. The major strength of this study is its population-based design, allowing unbiased estimates of DR incidence and progression. Additional strengths include standardised, masked grading of fundus photographs by trained graders, a low proportion of unreadable images, and standardised assessment of risk factors.

## CONCLUSIONS

In conclusion, over a 12-year period, the cumulative incidence of VTDR was relatively low (4%), but the progression to VTDR among individuals with baseline DR was substantial (17%). Apart from elevated BP and poor glycaemic control, younger age and Indian ethnicity were associated with incident VTDR. These findings support the potential value of risk-stratified screening, with earlier initiation for those at higher risk of incident VTDR and closer monitoring for those at risk of progression. Subgroups such as Indian individuals, those with poor glycaemic control, and long-standing diabetes may benefit from more frequent retinal examinations, while those with stable metabolic control may require less frequent screening. Further modelling studies are needed before recommending changes to current screening intervals.

## Additional material


Online Supplementary Document
