# Supplementary material for: Long-term incidence and progression of vision-threatening diabetic retinopathy in Asian populations
Source: J Glob Health. 2026 Jun 19;16:04174. doi: 10.7189/jogh.16.04174 (PMC13280866; doi:10.7189/jogh.16.04174)
Supplement: Online Supplementary Document [file jogh-16-04174-s001.pdf]

Supplement to: Koh BMQR, Chong C, Tan G, Cheng CY, Sabanayagam C. Long-term incidence and progression of vision-threatening diabetic retinopathy in Asian populations. J Glob Health. 2026;16:04174.

Table S1. Additional risk factors associated with incident VTDR in multivariable regression analysis

	Number at risk	Number of Incident VTDR (%)	Age, sex-adjusted RR (95% CI)	Multivariable adjusted RR (95% CI)*
Education level				
Below primary education	234	5 (2.1)	Reference	
Primary education	495	19 (3.8)	1.26 (0.44-3.55)	
Secondary/above education	448	21 (4.7)	1.37 (0.48-3.88)	
Current smoking				
No	1020	41 (4.0)	Reference	
Yes	157	4 (2.5)	0.46 (0.16-1.35)	
BMI, kg/m^2	1177	45 (3.8)	0.95 (0.89-1.01)	0.98 (0.92-1.05)
Systolic BP, mm Hg	1177	45 (3.8)	1.01 (1.00-1.03)	**1.01 (1.00-1.03)**
Total cholesterol, mmol/L	1177	45 (3.8)	1.39 (1.12-1.72)	1.14 (0.91-1.42)
HDL cholesterol, mmol/L	1177	45 (3.8)	0.88 (0.29-2.66)	0.78 (0.22-2.79)
Anti-diabetic medication use				
No	545	27 (5.0)	Reference	Reference
Yes	632	18 (2.8)	0.66 (0.36-1.21)	0.56 (0.29-1.06)

RRs were calculated using modified Poisson regression, with a robust error variance.

*Adjusted for age, sex, ethnicity, duration of diabetes, systolic BP and HbA1c

Table S2. Risk factors associated with Incident VTDR in sex-specific analysis

	Female (N = 578)		Male (N = 599)	
	Multivariable adjusted RR (95% CI)*	P value	Multivariable adjusted RR (95% CI)*	P value
Age, years	0.92 (0.86-0.98)	**0.011**	0.97 (0.92-1.03)	0.4
Ethnicity				
Malay	Reference		Reference	
Indian	2.33 (0.88-6.17)	0.1	1.90 (0.74-4.88)	0.2
BMI, kg/m^2	0.97 (0.89-1.06)	0.5	1.01 (0.91-1.12)	0.9
Systolic BP, mm Hg	1.02 (1.00-1.03)	**0.014**	1.01 (0.98-1.03)	0.6
Total cholesterol, mmol/L	1.00 (0.74-1.34)	1.0	1.26 (0.94-1.70)	0.1
HDL cholesterol, mmol/L	0.78 (0.09-6.72)	0.8	1.47 (0.28-7.76)	0.7
Duration of diabetes, years	1.06 (0.98-1.14)	0.2	1.08 (1.03-1.14)	**0.002**
HbA1c, %	1.58 (1.33-1.88)	**<0.001**	1.37 (1.15-1.62)	**<0.001**
Anti-diabetic medication use				
No	Reference		Reference	
Yes	0.27 (0.09-0.78)	**0.016**	1.03 (0.43-2.47)	1.0

RRs were calculated using modified Poisson regression, with a robust error variance.

The bold values denote p<0.05

*Adjusted for age, ethnicity, duration of diabetes, systolic BP and HbA1c

Table S3. Risk factors associated with Incident VTDR in ethnicity-specific analysis

	Malays (N = 499)		Indians (N = 678)	
	Multivariable adjusted RR (95% CI)*	P value	Multivariable adjusted RR (95% CI)*	P value
Age, years	0.93 (0.86-1.01)	0.1	0.97 (0.93-1.02)	0.2
Gender				
Male	Reference		Reference	
Female	1.00 (0.34-2.97)	1.0	1.07 (0.50-2.26)	0.9
BMI, kg/m^2	0.99 (0.90-1.09)	0.8	0.98 (0.90-1.07)	0.6
Systolic BP, mm Hg	1.03 (1.00-1.05)	**0.039**	1.01 (0.99-1.02)	0.3
Total cholesterol, mmol/L	0.83 (0.54-1.27)	0.4	1.34 (1.01-1.78)	**0.040**
HDL cholesterol, mmol/L	0.55 (0.03-9.05)	0.7	0.94 (0.24-3.76)	0.9
Diabetic duration, years	1.10 (1.01-1.20)	**0.036**	1.07 (1.01-1.12)	**0.023**
HbA1c, %	1.52 (1.28-1.82)	**<0.001**	1.49 (1.27-1.74)	**<0.001**
Anti-diabetic medication use				
No	Reference		Reference	
Yes	0.36 (0.12-1.03)	0.1	0.76 (0.33-1.77)	0.5

RRs were calculated using modified Poisson regression, with a robust error variance.

The bold values denote p<0.05

*Adjusted for age, sex, duration of diabetes, systolic BP and HbA1c

Table S4. Characteristics of Included and Excluded Participants

	Excluded (N = 1518)	Included (N = 1177)	P value
Age, years	62.7 (10.3)	57.8 (9.2)	**<0.001**
Gender, %			
Female,	777 (51.2)	578 (49.1)	0.3
Male	741 (48.8)	599 (50.9)	
Ethnicity, %			
Malay	695 (45.6)	499 (42.4)	0.1
Indian	823 (54.2)	678 (57.6)	
Education level, %			
Primary or below education	1124 (74.0)	732 (62.2)	**<0.001**
Secondary/above education	390 (25.8)	445 (37.8)	
Current smoking, yes, %	203 (13.4)	154 (13.1)	0.8
Alcohol consumption, yes, %	99 (6.6)	93 (7.9)	0.2
Hypertension, yes, %	1253 (82.8)	858 (73.2)	**<0.001**
BMI, kg/m^2	27.0 (5.0)	27.8 (4.8)	**<0.001**
Systolic BP, mm Hg	148.9 (23.9)	141.4 (19.6)	**<0.001**
Total cholesterol, mmol/L	5.3 (1.4)	5.2 (1.1)	**0.02**
HDL cholesterol, mmol/L	1.2 (0.3)	1.1 (0.3)	**<0.001**
Duration of diabetes, years	8.8 (9.9)	5.0 (6.4)	**<0.001**
HbA1c, %	7.7 (1.8)	7.3 (1.6)	**<0.001**

p values represent the difference in characteristics by inclusion status based on Student's *t*-Test or χ^2 test as appropriate for the variable
